# Utility of Tubular Retractors Augmented with Intraoperative Ultrasound in the Resection of Deep-seated Brain Lesions: Technical Note

**DOI:** 10.7759/cureus.4272

**Published:** 2019-03-19

**Authors:** Samer G Zammar, Jared Cappelli, Brad E Zacharia

**Affiliations:** 1 Neurosurgery, Penn State Health Milton S. Hershey Medical Center, Hershey, USA

**Keywords:** brain tumor, ultrasound, tubular retractor, deep brain lesions

## Abstract

Traditional brain retraction has been associated with significant damage to the healthy brain tissue particularly when attempting to expose a deep-seated lesion of the brain. Tubular retractors tend to provide a surgical corridor to treat these lesions while minimizing the extent of retraction on the brain. Intraoperative ultrasound can be used as a handy adjunct in maximizing the safe resection primarily by identifying the entry point, visualizing the lesion, and providing real-time feedback on the extent of resection. The authors provide a technical note with case illustrations on the use of tubular retractors augmented with intraoperative ultrasound to ensure a maximal safe resection of deep-seated brain lesions.

## Introduction

Minimal access neurosurgery is becoming increasingly common. In particular, the use of tubular retractors has recently gained traction as an option to decrease morbidity during surgical resection of deep-seated brain lesions. As these approaches become more physically constrained, accurate intraoperative localization and navigation become increasingly critical. Ultrasound is a fast, inexpensive, real-time tool that has a long history in neurosurgery, but has yet to be fully incorporated into tube-based neurosurgery [1, 2]. We report our experience with the use of ultrasound in tubular surgery and provide technical details regarding augmentation of standard tubular-based surgery techniques with a modified ultrasound probe integrated with the NICO Brainpath.

## Technical report

Patient selection

In patients with deep-seated brain lesions the optimal management strategy is dependent on a multitude of factors including lesion location, size, tissue characteristics, patient comorbidities and the need for a histologic diagnosis. We give consideration to the full range of treatment options for these lesions including traditional open surgical resection, tubular-based surgical resection, radiosurgery and/or laser interstitial thermal therapy. Typically, for patients with lesions between 1.5 cm and 3.5 cm in whom a natural corridor does not exist (i.e., interhemispheric, supracerebellar-infratentorial), who are symptomatic or require a histologic diagnosis we consider tubular-based surgical resection. In the United States, there are two commercially available system: ViewSite Brain Access System (Vycor Medical Inc., Boca Raton, FL) and BrainPath (Nico Corporation, Indianapolis, IN). These systems are primarily composed of a transparent outer cannula and an obturator that allows the surgeon to separate the white matter tract with blunt dissection. We currently utilize the NICO Brainpath as this facilitates a parafascicular approach, although the principles can be applied to either system.

Operative technique

Pre-operative Planning

In addition to routine neurosurgical imaging (i.e., magnetic resonance imaging (MRI) and/or computed tomography (CT)), we routinely obtain diffusion tensor imaging (DTI) sequences and perform 3D-tractography to identify the location of critical white matter pathways and define a trajectory, which spares these pathways. Attention is paid to the density of the lesion, cystic components and the relationship to the ventricular system, as these are particularly useful landmarks during intraoperative ultrasonography.

Operating Room Setup

Ideally, the patient should be positioned in a way so that the cavity is perpendicular to the floor. This facilitates a straightforward cannulation, which improves surgical ergonomics and allows for retention of irrigation that facilitates ultrasonography. The exact position of the patient is largely dependent on the location of the lesion and the preplanned trajectory approach, determined preoperatively by the surgeon and confirmed intraoperatively prior to incision planning.

We utilize an exoscopic visualization system (VITOM-90® from Karl Storz Endoscopy, Tuttlingen, Germany). We feel that exoscopic visualization improves ergonomics, as it dissociates the operator from the oculars. The procedure, however, can be performed with a traditional surgical microscope. In the majority of circumstances, we avoid hyperventilation and preoperative mannitol to avoid excessive brain relaxation, as some brain pressure is required to provide resistance during cannulation. The approach is image guided and appropriate neuronavigation is required. We utilize the Stealth S8 (StealthStation™ S8 surgical navigation system from Medtronic, Minneapolis, MN) and rigidly fix the head in Mayfield fixation prior to optical registration.

Craniotomy and Sulcal Identification

After creation of an appropriate trajectory and starting point, a craniotomy is planned. A craniotomy ~ 4-6 cm in diameter is completed. While a smaller craniotomy can be utilized, adequate room to “wand” the tube and avoid collision with the bone edge is recommended. The dural opening, on the other hand, should be limited to the diameter of the retractor (~ 15 mm) to provide a tight seal around the tubular retractor. The goal is a transsulcal parafascicular approach parallel to white matter pathways. This requires identification of an appropriate sulcus for entry. This is initially accomplished by evaluation of preoperative contrast-enhanced imaging and confirmed with intraoperative neuronavigation.

It is at this stage, prior to dural opening, that we perform our initial ultrasonography utilizing a (ProSound Alpha 7 ultrasound system, Hitachi-Aloka, Wallingford, CT). We utilize a standard brain probe as this provides the optimal image. We take particular note of the lesion location relative to the craniotomy, depth from the surface and previously identified ventricular landmarks and the echogenicity (i.e., lesion brightness). We then utilize standard and flow-enhanced ultrasound to identify an appropriate sulcus within the craniotomy flap. The dural opening can then be tailored immediately over the sulcus.

Cannulation and Lesion Identification

After dural opening the sulcus is identified grossly and the exoscope is brought into the field. The sulcus is then sharply opened with an arachnoid knife, microscissors and expanded with microforceps and dissectors. Only 1-1.5 cm of the sulcus needs to be opened and it typically does not need to be opened to the depth of the sulcus. Utilizing image guidance and the previously planned trajectory, the retractor can be advanced through the sulcus with the assistance of gentle irrigation. For most lesions we prefer a surface cannulation. By cannulating just above the lesion we are able to delineate the boundary between the normal surrounding brain tissue and the lesion.

It can be quite disconcerting when first using this technique to remove the obturator and visualize normal appearing white matter. This is, in our opinion, the most valuable use of the ultrasound technique. As the diameter of the NICO Brainpath is 13.5 mm, it cannot accommodate a standard ultrasound probe. The burr hole probe can be used, but the view is typically obscured by the tube and quite difficult to interpret. A standard brain probe can be utilized adjacent to the tube, but this requires significant additional space within the craniotomy defect and also suffers from tube artifact. A specially designed probe (BrainPath probe, Hitachi-Aloka, Twinsburg, OH) compatible with a tubular retractor system has overcome these limitations and afforded accurate and robust intraoperative ultrasound.

At this stage we utilize saline irrigation to fill the tube and bring in the 14- to 22-mm BrainPath ultrasound probe. If the cannulation was accurate, one will identify the lesion immediately subjacent to the tube. The tube edges and a small amount of white matter can also be identified. Gentle dissection can then proceed to grossly identify the lesion. If the lesion is not identified, one can utilize a “wanding” technique to change the angle of the tube while under ultrasonographic visualization. Often times this will reveal the lesion slightly off the original trajectory. The tube can then be angled appropriately. If the trajectory is significantly off, it may be necessary to remove the tube and recannulate.

Resection and Evaluation of Extent of Resection

After the lesion has been identified, two-handed surgical technique using a combination of suction, bipolar cautery and gentle dissection is utilized to resect the lesions. Larger lesions frequently require piecemeal resection. Given the limited diameter of the tube, use of single-shaft instruments facilitates microsurgical resection. We have utilized single shaft instruments designed for endoscopic endonasal use (Storz, Sephernia, Tuttlingen, Germany) and feel these are quite helpful. Generally due to the limited window provided by the BrainPath cannulation device, utilization of certain instruments, such as an ultrasonic aspirator, is difficult. The NICO Myriad is a single shaft side-cutting aspirator device, which facilitates resection of firmer lesions through the limited diameter of the tube. The tissue can be collected in a trap and utilized for pathologic analysis. Following resection of the lesion the ultrasound is re-introduced and the cavity inspected. Again, the tube can be gently manipulated to provide a circumferential view of the cavity. We try using ultrasound prior to instillation of hemostatic material as this can obscure the ultrasonographic image. If additional lesion is identified, it can be resected and this procedure repeated.

When resection is completed, immaculate hemostasis is obtained in the standard fashion and the tube is gently backed out under direct visualization. The dura is then closed and the bone flap is secured in place. The wound is closed in the standard fashion.

Case examples

Case 1

**Figure 1:**
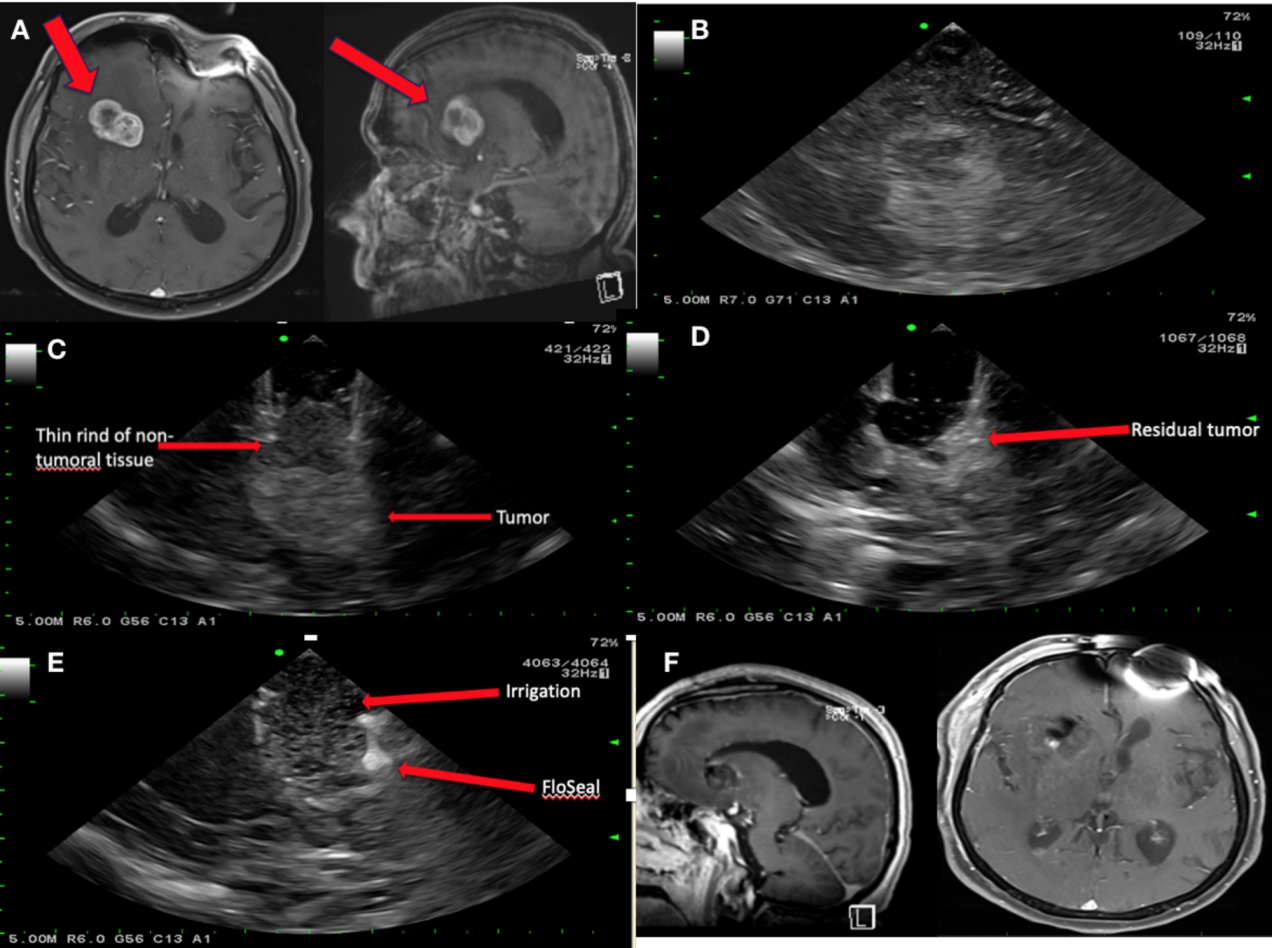
Magnetic resonance imaging (MRI) and ultrasound pre- and post-resection using BrainPath. (A) A T1-weighted MRI with contrast (axial sequence on the left and sagittal sequence on the right) showing a right periventricular enhancing lesion. (B) The use of intraoperative ultrasound after the craniotomy is performed to identify the tumor. (C) The use of intraoperative ultrasound to visualize the thin rind of non-tumoral tissue that can obscure the tumor as well as the tumor which is located deep to that rind. (D) The use of intraoperative ultrasound to show gross residual tumor. (E) The use of intraoperative ultrasound to show gross total resection, the irrigated surgical cavity as well as the applied hemostatic matrix. (F) A T1-weighted postoperative MRI with contrast (sagittal sequence on the left and axial sequence on the right) showing no residual tumor.

A 69-year-old gentleman with a known history of non-small cell lung cancer presented with one-week onset of left-sided facial droop, dysarthria, and shuffling gait. An MRI demonstrated a right frontal periventricular enhancing lesion (Figure 1A). Due to its deep location and symptomatic presentation, the decision was made to use the BrainPath augmented with ultrasound via a right frontal parafascicular approach to resect the lesion. Figure 1B shows the use of ultrasound after the craniotomy to identify the lesion. After appropriate sulcal identification and docking of the system, ultrasound was used again to visualize the lesion, ensure appropriate docking, and detect the thin rind of non-tumoral tissue that can obscure the tumor (Figure 1C). After resection was completed, the ultrasound was reintroduced and demonstrated a small amount of residual tumor (Figure 1D), thus the resection was continued until no obvious residual was demonstrated on ultrasound. After the resection was satisfactory, the wound was irrigated and FloSeal (FloSeal Hemostatic Matrix from Baxter International Inc., Deerfield, IL) was applied, as shown in Figure 1E. Post-operative MRI showed adequate resection and the patient was discharged home on post-operative day 2 with plans for adjuvant gamma knife radiosurgery (Figure 1F).

Case 2

**Figure 2:**
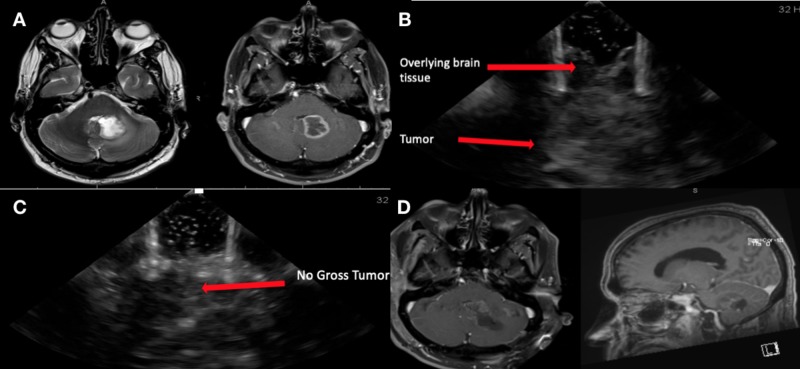
Magnetic resonance imaging (MRI) and ultrasound pre- and post-resection using BrainPath. (A) An axial T2-weighted MRI on the left and T1-weighted MRI with contrast on the right showing a T2 hyperintense and ring enhancing lesion in the cerebellum compressing the 4^th^ ventricle. (B) The use of intraoperative ultrasound after craniotomy is performed to identify the tumor as well as the brain tissue overlying the tumor. (C) The use of intraoperative ultrasound to show gross total resection. (D) A T1-weighted postoperative MRI with contrast (axial sequence on the left and sagittal sequence on the right) showing no residual tumor.

A 52-year-old gentleman with a long-term history of smoking presented with headache and dizziness and was found to have a 2.7-cm cerebellar lesion on MRI (Figure 2A). BrainPath augmented with ultrasound was used to resect the lesion and obtain a histologic diagnosis. The ultrasound was able to identify the tumor and overlying brain tissue, as shown in Figure 2B. After maximal resection was attempted, ultrasound did not show gross tumor residual (Figure 2C). Postoperative MRI showed adequate resection (Figure 2D).

The pathology was consistent with metastatic adenocarcinoma of the lung and the patient was discharged home on post-operative day 2 with plans for adjuvant gamma knife radiosurgery.

## Discussion

Resurgence of tubular-based retractors/surgery

While the use of traditional retractors in brain surgery provides a good surgical corridor, these retractors can stress and damage the parenchyma and reduce tissue perfusion by disrupting the blood vessels and blood-brain barrier [3-5]. Tubular retractors emerged in an attempt to minimize retraction on the brain during the resection of deep brain lesions. Tubular retractors are thought to decrease the pressure on healthy brain tissue by distributing the pressure over the outer surface area of the cylinder, thus minimizing potential neurological complications [6]. Since 1987, tubular retractors have been shown to be useful in resecting deep-seated and centrally located intra-axial brain lesions with satisfactory surgical outcomes [7], but only recently have they gained traction, given improvements in device, optical and imaging technology.

Tubular retractors have been used in the treatment of intracerebral hemorrhage and vascular lesions. Several studies have demonstrated the efficacy of this technique in the evacuation of deep brain hematomas, access ruptured periventricular aneurysms and cerebral cavernous malformations, and resection of deep-seated brain tumors with minimally invasive trans-sulcal parafascicular resection [8-15].

General uses of intraoperative ultrasound in neurosurgery

In 1982, Chandler et al. described the first use of intraoperative ultrasound in 21 neurosurgical procedures which included tumor resection, arteriovenous malformation resection, and ventricular catheter placement [16]. Since then, real-time ultrasonography has been widely used as an effective adjunct in neurosurgical procedures to help with accurate lesion localization and treatment [2]. In brain tumor surgery, maximizing extent of resection while minimizing surgical morbidity remains of paramount importance. Intraoperative neuronavigation is routinely incorporated into cranial neurosurgery and affords accurate lesion localization, but its accuracy is limited by brain shift secondary to intraoperative brain swelling, cerebrospinal fluid (CSF) drainage and tissue manipulation. Consequently, the necessity of having a real-time imaging tool intraoperatively became of utmost importance. While intraoperative MRI [17], cone-beam CT and other modalities have been used for this purpose, ultrasound has remained attractive due to its relatively low cost and immediate feedback. Intraoperative ultrasound has proven to be useful in intraoperative navigation, assessment of resection, as well as compensation and monitoring of brain shift [1,2,18-20].

## Conclusions

The use of tubular retractors has recently gained traction as an option to decrease morbidity during surgical resection of deep-seated brain lesions. As these approaches become more physically constrained, accurate intraoperative localization and navigation become increasingly critical. Ultrasound is a fast, inexpensive, real-time tool that can optimize the efficacy and safety of these operations by aiding sulcal identification, lesion localization and maximize extent of resection.
